# Chemical Composition and Biological Activities of Mono- and Heterofloral Bee Pollen of Different Geographical Origins

**DOI:** 10.3390/ijms18050921

**Published:** 2017-04-27

**Authors:** Jucilene Silva Araújo, Emerson Dechechi Chambó, Maria Angélica Pereira de Carvalho Costa, Samira Maria Peixoto Cavalcante da Silva, Carlos Alfredo Lopes de Carvalho, Leticia M. Estevinho

**Affiliations:** 1 Centro de Ciências Agrárias, Ambientais e Biológicas, Universidade Federal do Recôncavo da Bahia, Cruz das Almas 44380-000, Bahia, Brazil; jucilenearaujo15@hotmail.com (J.S.A.); mapcosta@ufrb.edu.br (M.A.P.d.C.C.); samypeixoto@yahoo.com.br (S.M.P.C.d.S.); calfredo@ufrb.edu.br (C.A.L.d.C.); 2 Instituto de Natureza e Cultura, Universidade Federal do Amazonas, Benjamin Constant 69630-000, Amazonas, Brazil; chamboed@gmail.com; 3 Departamento de Biologia e Biotecnologia, Escola Superior Agrária, Instituto Politécnico de Bragança, Bragança 5301-855, Portugal; 4 Centro de Biologia Molecular e Ambiental, Universidade do Minho, Campus de Gualtar, Braga 4710-057, Portugal

**Keywords:** antioxidant, enzyme inhibitory, fatty acid, phenolic content

## Abstract

Recent research shows variations in pollen chemical constituents and, consequently, in their therapeutic properties. Mono and multifloral bee pollen extracts were investigated for antioxidant and enzyme inhibitory activity properties, phenolic compounds and fatty acid composition. Generally, *Eucalyptus* spp. and multifloral extracts exhibited potent inhibitory activity against α-amylase, acetylcholinesterase, tyrosinase, lipoxygenase, lipase and hyaluronidase. On the other hand, *Miconia* spp. demonstrated higher antihemolytic activity. *Cocos nucifera* and *Miconia* spp. extracts exhibited important antioxidant properties in the different assays (ABTS, DPPH, β-carotene/linoleic acid and reducing power). Moreover, these extracts had greater amounts of total phenols and flavonoids in comparison to others. The increase in antioxidant activity (decrease in EC_50_ values) was accompanied by an increase in the amount of total phenols in the extracts. The pollen extracts contained linoleic acid and α-linolenic acid as major fatty acids, followed by palmitic acid, and oleic acid. In this study, differences were observed in both chemical constituents and biological activities of the samples related to the geographical and botanical origin of bee pollen.

## 1. Introduction

Bee pollen has a highly complex and nutritive chemical composition [1], but its constituents vary within a minimum and maximum range of values, especially due to geographical and botanical origin, as well as edaphoclimatic conditions [2,3,4,5].

This beehive product is considered a healthy food, with a wide range of beneficial effects for human health, including protection against depression and anxiolytic properties, memory improvement and antiepileptic effect as well as decrease on the rate of bone loss due to osteoporosis on mice [6,7]. Antimicrobial, antifungal, antioxidant, anti-inflammatory and liver protection properties have also been claimed in diverse studies using bee pollen from diverse geographical and botanical settings [8,9].

Lipid mediators play a key role in immune regulation and homeostasis maintenance of the human organism. Indeed, the increased production of tyrosinase is associated with augmented intracellular dopamine production, followed by the induction of melanin formation, causing cell death [10]. On the other hand, high levels of lipase disrupt the functioning and internal structure of the pancreas, whereas hyaluronidase contributes to local tissue destruction associated with inflammatory process [11]. Also, the aberrant increase activity of acetylcholinesterase is associated with the etiopatogenicity of Alzheimer’s disease [12].

As such, the use of enzyme inhibitors for the manipulation of lipid mediator signaling has great therapeutic potential. Although many enzyme inhibitors for the treatment of diseases are produced artificially, research shows that artificial inhibitors cause side effects such as liver damage and gastrointestinal disorders [11,12]. On the other hand, active compounds in herbal medicines are in a state of biological equilibrium with other compounds and, consequently, do not accumulate in the body, having fewer or no side effects [6].

In this context, bee pollen can be a promising alternative to chemical medicines for the prevention of some diseases. Its efficiency in the anti-inflammatory process through its ability to inhibit the hyaluronidase enzyme [13] and to improve overall symptoms of inflammatory diseases with no side effects has been proven [14].

Indeed, recent studies have been intensified in order to obtain additional information on chemical composition and therapeutic properties of pollen [15,16,17,18]. These investigations show variations in the phenolic content of pollen and in its biological activities, which has led to further investigations of these differences within and between pollen types, instead of assuming that all have the same health benefits.

We tested the hypothesis regarding differences in the chemical composition and biological activity of pollen, especially due to geographical and botanical origin. This study compared the chemical composition of bee pollen mono- and heterofloral of different locations. We also evaluated and compared their antioxidant activity and enzyme inhibition properties (α-amylase, acetylcholinesterase, tyrosinase, lipoxygenase, lipase and hyaluronidase). As far as we know, no other study has investigated the effect of this natural product on the inhibition of the enzymes examined here.

## 2. Results

### 2.1. Fatty Acid Composition

In this study, eleven fatty acids were identified and quantified; their number of carbons ranged from C4 to C18 (Table 1). The linoleic acid (PUFA, C18:3n3, ranging from 1.471 to 1.758 g/100 g of bee pollen) and the α-linoleic acid (PUFA, C18:2n6c, ranging between 0.657 and 0.970 g/100 g of bee pollen) were the fatty acids (FA) detected in larger quantities. Palmitic (SFA, C16:0) and oleic (PUFA, C18:1n9, PUFA) acids were also present in high quantities and their concentrations significantly differed among the extracts (*p* < 0.05). Extracts S4, S5 and S7 had significantly higher quantities of C16:0. On the other hand, extract S7 had greater amounts of C18:1n9, followed by S6, S5 and S4 (the concentration obtained for these three samples did not differ statistically).

Saturated fatty acids (SFA) ranged from 0.655 ± 0.011 to 1.345 ± 0.033; Monounsaturated fatty acids (MUFA) ranged from 0.328 ± 0.024 to 0.0950 ± 0.028; while the values obtained for Polyunsaturated fatty acids (PUFA) were between 1.861 ± 0.060 and 2.758 ± 0.162. For these three parameters, significantly higher values were obtained for extract S7.

The ratio PUFA: SFA was significantly superior (*p* < 0.05) in extract S8 (3.823 ± 0.046), followed by S7, S2, S3 (these three did not differ statistically). Regarding the ratio n6:n3, no statistical differences were found among the different samples.

The extracts S4, S5, S6 and S7 had a significantly higher (*p* < 0.05) thrombogenic index (TI) when compared to the others. The atherogenic index (AI) also varied among samples, ranging between 0.066 ± 0.04 (extract S8) and 0.102 ± 0.010 (extract S7).

### 2.2. Antioxidant Activities

Antioxidant activities of pollen extracts were evaluated by a free radical scavenging assay (ABTS and DPPH), a β-carotene bleaching assay (BCB) and ferric reducing power (FRP).

Generally, pollen extracts S1–S5 showed the highest activity with lower values of EC_50_. The highest ABTS scavenging activity was observed in pollen extract S4, followed by S3 and S5. However, the highest DPPH, BCB assay and FRP inhibition were caused by extract S1 and S2, followed by extracts S3–S5. The EC_50_ values for extracts with smaller activities (higher EC_50_ values) were up to five fold higher than for those with better antioxidant activities. The pollen extract S7 exhibited a lowest activity with respect to both ABTS and DPPH, while extract S6 presented the lowest activity in the BCB assay and FRP (Table 2).

**Figure 1:**
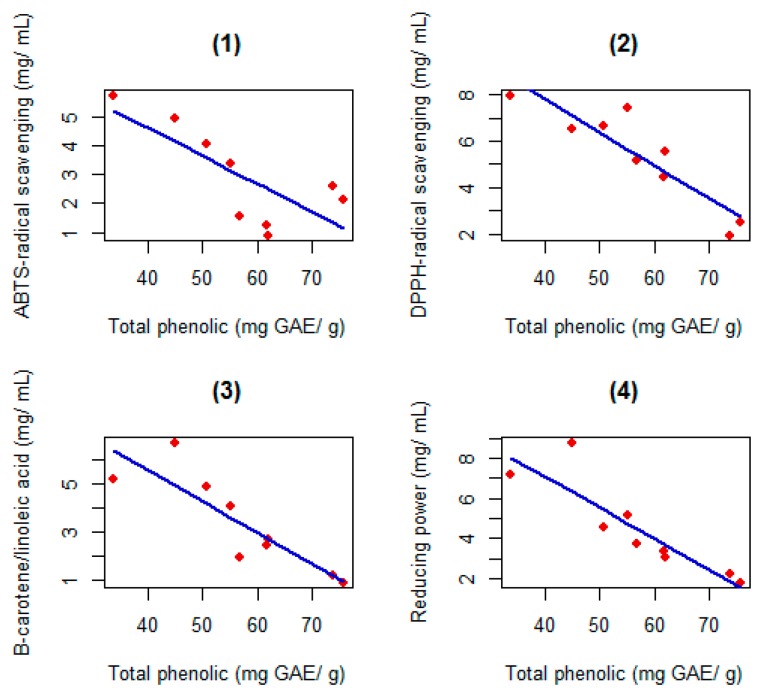
Regression equations estimated for the four evaluation methods of the antioxidant activity in relation to the amounts of total phenols. (**1**) *y* = −0.0965*x* + 8.4587 (*R*^2^ = 57%, *p* < 0.01); (**2**) *y* = −0.1429*x* + 13.5332 (*R*^2^ = 82%, *p* < 0.01); (**3**) *y* = −0.1294*x* + 10.7458 (*R*^2^ = 76%, *p* < 0.01); (**4**) *y* = −0.1539*x* + 13.2256 (*R*^2^ = 79%, *p* < 0.01).

The regression equations relating antioxidant activity with total phenolic showed a linear decrease in EC_50_ values as the amount of total phenols increased (Figure 1), for all methods.

### 2.3. Total Phenolic and Flavonoid

**Figure 2:**
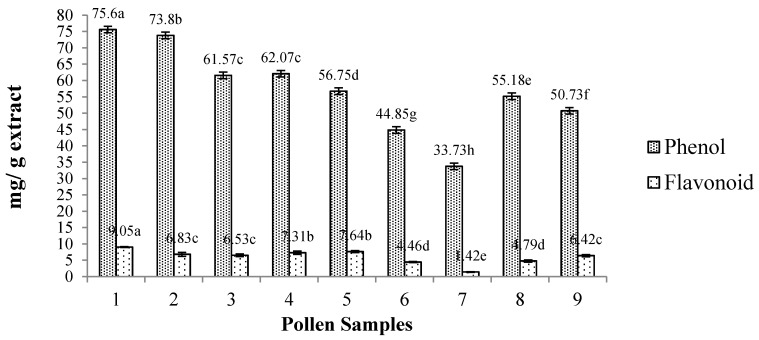
Concentration of total phenolics and flavonoids of the bee pollen extracts (mean ± SD). Different letters represent significant differences (*p* < 0.05). Phenols are expressed as mg gallic acid equivalents/g extract (GAE/g extract); while flavonoids are expressed as mg quercetin equivalents/g extract (QE/g extract). Extracts: S1—*Cocos nucifera*; S2—*Cocos nucifera*; S3—*Miconia* spp.; S4—*Miconia* spp.; S5—*Miconia* spp; S6—*Spondias* spp.; S7—*Myrcia* spp.; S8—*Eucalyptus* spp.; S9—*Eucalyptus* spp.

The amounts of total phenols and flavonoids of pollen extracts are shown in Figure 2. The total phenolic content of the pollen extracts ranged from 33.73 to 75.60 mg GAE/g and for flavonoids, from 1.42 to 9.05 mg QE/g of bee pollen extract. Higher amounts of total phenols were found in extract S1, followed by S2. Pollen extract S1 had superior amounts of flavonoids, followed by extracts S4 and S5 (no difference). Extract S7 had the lowest amounts of total phenols and flavonoids.

### 2.4. Enzyme Inhibitory Activities

The pollen extracts were evaluated regarding the inhibitory activities of: α-amylase (α-AMY), acetylcholinesterase (AChE), tyrosinase (TYR), lipoxygenase (LOX), lipase (LIP), hyaluronidase (HYAL). The anti-hemolytic activity (AHA) was also assessed.

Extracts S8 and S9 had higher inhibition activities for α-AMY and TYR and did not differ statistically. Extract S7 had greater inhibitory activity for AChE, followed by extracts S8 and S9, which were similar. Extracts S6 and S7 did not differ and had greater inhibitory activities against LOX. Regarding the inhibition of LIP, extract S6, followed by S7 and S9, was the most efficient. Extract S1, followed by S2, had greater inhibitory action against HYAL. There was greater inhibitory activity for AHA in extract S4. Several samples exhibit higher inhibition activity than the positive controls (S8 and S9 to α-AMY inhibition and S6, S7, S8 and S9 to LOX inhibition) (Table 3). 

**Figure 3:**
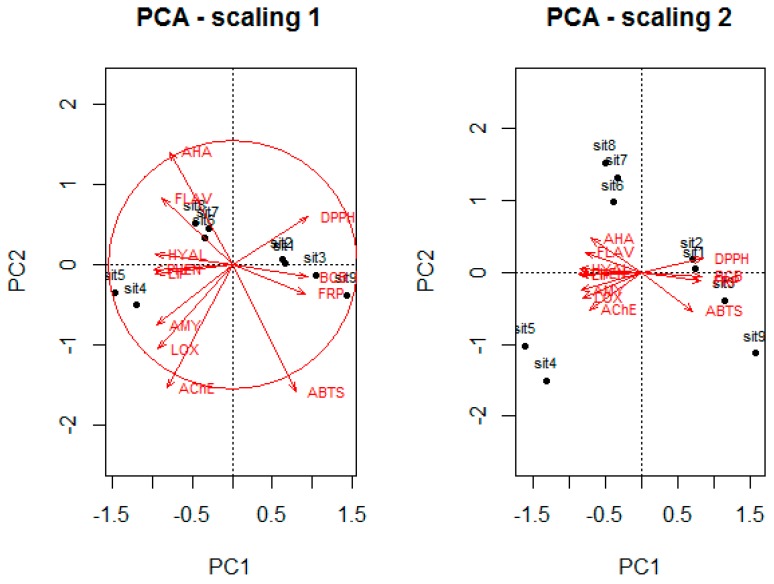
PCA biplots of total phenolic and flavonoid, enzyme inhibitory and antioxidant activities. The bottom and left-hand scale regard the bee pollen extract, while the top and right-hand scale are for the variables. PC1 and PC2: 80% and 11%, respectively, of proportion explained.

In Figure 3, the variance proportion accounted for by the first two axes is 92%. This high value indicates that the first two components are sufficient to extract the most relevant information from the data. Variables AHA, AChE and ABTS had the highest contributions to the ordering and can be interpreted with greater confidence.

Biplot scaling 1 reveals a gradient from left to right, starting with a group formed by extracts S4 and S5 displaying the highest values of AMY, LOX, AChE, LIP, HYAL and total phenolic (PHEN), and the lowest values for DPPH. The second group of extracts (S6, S7 and S8) possesses the highest concentration of flavonoid (FLAV) and AHA and the lowest ABTS. Some very similar extracts (S1, S2, S3 and S9) constitute a third group that has the highest values of EC_50_ detected using all methods for evaluating antioxidant capacity and the lowest for enzyme inhibitory activities, i.e., PHEN and FLAV.

The variables organized into groups are displayed in a scaling 2 biplot. The left part of this biplot reveals that PHEN, FLAV and enzyme inhibitory activities are very highly positively correlated. On the other hand, these variables are very highly yet negatively correlated with another group including the methods to detect the antioxidant activities.

## 3. Discussion

According to previous studies [3,5,6], small differences in the chemical composition of bee pollen from a particular botanical taxon are common and may be attributed to variations in the geographical origin. However, the major differences in the chemical constitution of this beehive product are mainly attributed to botanical origin [2,4,19].

Despite this variability, our extracts showed high amounts of total phenols and values, which are within the range reported in studies using samples from Poland and Brazil [15,20]. However, [8,16,17,21] observed smaller values in pollen from Portugal, Lithuania, Brazil and Algeria, respectively.

The concentration of flavonoids obtained for the samples is in agreement with the values reported by LeBlanc et al. [22] and Feás et al. [23]. However, Carpes et al. [24], who analyzed bee pollen from Brazil, obtained higher amounts of these compounds—ranging from 2.10 to 28.33 mg quercetin/g.

According to the literature [8], the antioxidant capacity of bee pollen must be approached using more than one methodology, due to the complexity and heterogeneity of the matrix. In this study, this activity was assessed using four methodologies (ABTS, DPPH, BCB and FRP) and all extracts presented low EC_50_ values, regardless of the method used.

Recent studies show that the antioxidant activity of bee pollen extracts is associated positively with the content of phenolic compounds [17,21,25]. On the other hand, this does not appear to be true for other biological activities [13,15]. The phenolic compounds comprise a group of different structures with several properties [26] and the number and the position of hydroxyls present in the molecule and steric effects determine their antioxidant activity [27]. It is possible that it is not the quantity of total phenolic compounds that results in increased antioxidant activity, but the presence and concentration of particular phenolic compounds. As such, the qualitative phenolic composition may play a more important role in the quantitative composition. Indeed, for example, Leja et al. (2007) [15], found greater total antioxidant activity in pollen extracts only by increasing the amounts of phenylpropanoids.

As far as the authors know, except regarding hyaluronidase and monoamine oxidase, the enzyme inhibition activity of bee pollen has not been studied. Here, all extracts showed some inhibitory activity against the enzymes under study, which are involved in the pathophysiological pathway of important health conditions.

Yildiz et al. [7] found that inhibition of the enzyme monoamine oxidase is related to the total content of phenolic compounds as well as the antioxidant capacity of bee pollen. Also, Pascoal et al. [13] observed a positive relationship between bee pollen polyphenols content and the percentage of inhibition of the hyaluronidase enzyme.

However, in this study, no association was observed between enzyme inhibition properties and polyphenol concentration. In fact, according to Markiewicz-Żukowska et al. [28], the possible contribution of other non-phenolic compounds to biological activity cannot be dismissed. Indeed, substances like fatty acids, phospholipids, phytosterols and organic carotenoid pigments—which are all present in bee pollen—have been claimed to possess important biological activities and their contribution to the health promoting effects should not be neglected [6].

The fatty acid composition was variable depending on the sample. Even so, the most prevalent were, for all extracts, α-linolenic (C18:3n3) and linoleic acid (C18:2n6c). These results are similar to those obtained in previous studies carried out using Italian bee pollen [29]. All the studied extracts presented PUFA/SFA ratios greater than 0.45 and n6/n3 ratios smaller than 4 (thresholds recommended by the World Health Organization). Similar results were reported by Estevinho et al. [3] for Portuguese bee pollen samples. PUFA have long been recognized as playing a role in decreasing serum triacylglycerol levels, blood pressure and insulin resistance and, therefore, the risk of cardiovascular events [30].

Among these health promoting fatty acids, linoleic acid (n6 class) has been described to have anti-atheratogenic action, while α-linolenic (n3 class) is appreciated for its anti-thrombogenetic effect [31]. Thus, as expected, the atherogenic and the thrombogenic indexes were low for all samples and similar to the reported values for other acclaimed beneficial foods for health.

## 4. Materials and Methods

### 4.1. Samples

The nine types of bee pollen were collected by beekeepers, from separate apiaries located in Bahia: Neópolis, Maraú, Valença, Canavieiras and Teixeira de Freitas. After the beekeepers dried the bee pollen, the samples were delivered to the laboratory and stored in the dark at room temperature (±20 °C).

In order to ascertain the botanical origin of bee pollen samples, 1000 pollen grains were counted and assessed following the Erdtman method [32]. Table 4 presents the samples investigated with their respective botanical and geographical origins.

Pollen types identification was performed using a reference collection of the authors and, whenever needed, specialized atlas and literature. The samples were categorized in terms of pollen frequency using the following classes: dominant pollen (DP, more than 45% of pollen grains counted), accessory pollen (AP, 16–45%), important isolated pollen (IIP, 3–15%) and occasional isolated pollen (OIP, 1–3%) [33].

### 4.2. Determination of Fatty Acids

For fatty acids determination, a flame ionization detection (GC–FID)/capillary column was used, following the method described by Human et al. [34] with slight modifications. An automatic Soxtec device (FOSS, Soxtec™ 2050, Höganäs, Sweden) was used for crude fat (CF) extraction [35]. The CF fraction was transesterified using MeOH in the presence of H_2_SO_4_ (Merck, Darmstadt, Germany) in order to obtain fatty acid methyl esters. Then, a portion of bee pollen containing 20 ± 0.5 mg of lipids was dissolved in 0.75 mL of *n*-hexane (Merck) and 0.1 mL of a solution containing 2 N KOH in MeOH (Merck, Germany) was added. This solution was mixed for 2 min using a vortex (Model Reax 2000, Heidolph, Schwabach, Germany) and dried over anhydrous Na_2_SO_4_ (Merck).

A BÜCHI Fat Determination System (AOAC International, Rockville, MD, USA) was used for fat determination. The extract was separated through gas chromatography in a capillary column (DB-WAX 30 m × 0.32 mm ID × 0.25 μm: stationary phase of 50% cyanopropylmethyl–50% phenylmethylpolysiloxane), using hydrogen as a carrier gas (flow rate set at 4.0 mL·min^−1^), at a pressure of 0.61 bar and split ratio of 1:40. The thermal gradient ranged from 170 to 240 °C at 3.5 °C min^−1^ and the injector and detector temperatures were 240 °C.

Following the direct extraction method, the homogenized material was weighed into the reaction vessel with the internal standard tridecanoic acid (accuracy of 0.1 mg). Fatty acid quantification was carried out by response factor using a 37 standard fatty acid mixture (SupelcoTM 37 Component FAME Mix) as a standard solution. The contents of fat, saturated, mono- and poly-unsaturated fatty acids (MUFA and PUFA) and the content of each individual fatty acid were automatically calculated by the software by mean of a pre-established factor. As final product, a layout with all fatty acids found, and the sums of the total SFA and unsaturated fats of the injected sample were obtained. The results are an average of four independent replicates.

The atherogenic index (AI), relating the sum of the main pro-atherogenic saturated fatty and the sum of the main unsaturated ones (regarded as anti-atherogenic), and the thrombogenic index (TI), showing the tendency to form blood clots, were calculated according to the equations given in [30].

### 4.3. Preparation of Pollen Methanolic Extracts (PME)

The extracts were prepared as previously described in detail [36]. The extract of dry pollen was stored in the dark and at room temperature to determine the total phenolic compounds, total flavonoids, antioxidant activity and effect on enzymes.

### 4.4. Total Phenols and Flavonoids, and Antioxidant Activity

These determinations were carried out using the Folin-Ciocalteau method described by Morais et al. [8] and Moreira et al. [37]. The content of total phenols was expressed as mg gallic acid (GAE) equivalents per g of bee pollen.

Determination of the total flavonoid content followed the procedures of Feás et al. [23]; results are expressed as mg of quercetin equivalents (QE) per g of pollen dried extract. In order to avoid possible interferences and considering the complex nature of bee pollen constituents, the antioxidant activity was determined using four methodologies, as recommended by Sakanaka and Ishihara (2008): evaluation of the effect of free radical DPPH blocker (2.2-diphenyl-1-picrylhydrazyl) based on the methodology advocated by Ferreira et al. [38]; evaluation of ferric reducing/antioxidant power according to the method described by Berker et al. [39]; inhibition of β-carotene bleaching (BCB) based on Ahn et al. [40], and determining the removal of ABTS radicals according to Miguel et al. [41].

### 4.5. Enzymatic Activities

#### 4.5.1. Lipase

To evaluate the activity of pancreatic lipase, we used the method described by Roh and Jung [42] with minor modifications, using *p*-Nitrophenyl Butyrate (*p*-NPB) (Sigma Chemical Co., St. Louis, MO, USA) as a substrate. We prepared solutions of porcine pancreatic lipase (EC 3.1.1.3) (PPL), Sigma Chemical Co., (1 mg/mL) in buffer of potassium phosphate 0.1 mM (pH 6.0), Merck). The solutions were stored at −20 °C. Pollen extracts dissolved in dimethyl sulfoxide (DMSO) (Merck) (final concentrations of 10, 5, 2.5, 1.25, 0.63, 0.31, 0.16 μg/ mL) or orlistat (Sigma Chemical Co.) at the same concentrations (positive controls) were pre-incubated at 30 °C. Afterward, 0.1 µL of PNPB (*p*-Nitrophenyl Butyrate) was added as a substrate. Following incubation for 5 min at 30 °C, the amount of *p*-nitrophenol released in the reaction was quantified using a UV spectrophotometer—Visible (Helios). DMSO was used as negative control and its activity with and without an inhibitor was also assessed.

#### 4.5.2. α-Amylase

Inhibition of α-amylase activity was evaluated according to the method reported by Gao and Kawabata [43] and Gao et al. [44]. The subtract starch azure (Sigma Chemical Co., 2.0 mg) was dissolved in Tris 50 mM-HCl buffer with a pH value of 6.9 containing 10 mM CaCl_2_ (both from Merck). Then, this solution was boiled at 100 °C for 5 min and pre-incubated for 10 min at 37 °C. Subsequently, to each test, a sample was added (at the final concentrations 1250, 1000, 750, 500, 250, 50, 10 μg/mL) dissolved in DMSO (50%) and 0.2 mL solution of α-amylase of pig pancreas (Sigma, A-6255; 2.0 U/mL; 50 mM Tri-HCl buffer with 10 mM CaCl_2_, pH of 6.9). Then, this was incubated for 10 min at 37 °C and the reaction was stopped by adding 0.5 mL of acetic acid (50%). The mixture was centrifuged at 2000 rpm for 5 min and the absorbance of the supernatant was measured at 595 nm. Acarbose (Sigma Chemical Co.) was the positive control.

Inhibitory activities (I%) of lipase and α-amylase were calculated using the equation: (I%) = 100 − ((B − b)/(A − a) × 100), in which *A* is the activity without inhibitor; a represents the negative control without inhibitor; B the activity with inhibitor; and b the negative control with inhibitor.

The EC_50_ value, the extract concentration providing 50% inhibition, was calculated by interpolation from the graph of I% against extract concentration.

#### 4.5.3. Acetylcholinesterase (AchE)

The inhibition of acetylcholinesterase was evaluated using the spectrophotometric method of Ellman et al. [45], with some modifications. We used acetylcholinesterase of Electrophorus electricus (electric eel Type-VI-S, Sigma Chemical Co.) and horse serum BChE (EC 3.1.1.8, Sigma Aldrich) as substrates in the reaction. To quantify the cholinesterase activity, we used acid 5.5-dithio-bis-(2-nitrobenzoic acid) (DTNB, Sigma Chemical Co.) [46]. Acetylcholine iodide hydrolysis was monitored throughout the formation of anion 5-tio-2-methyl nitrobenzoate (yellow color). The percentage inhibition of enzymes AChE/ BChE was determined by comparing the rates of the sample reactions (final concentrations of 1000, 750, 500, 250, 50, 10, 1.5 μg/mL) with those of the blank (phosphate buffer, with a pH of 8, and ethanol) using the formula (E − S)/E × 100, in which E is the activity of enzyme without test sample, while S is the activity of enzyme with test sample. The extract concentration providing 50% inhibition (EC_50_) was calculated by interpolation from the graph of I% against extract concentration. Eserine (Sigma Chemical Co.) was used as a reference.

#### 4.5.4. Tyrosinase

The inhibition of the activity of this enzyme was evaluated following the reported by Orhan et al. [47], with slight modifications. On a microplate of 96 wells (Multiskan™ GO Microplate Spectrophotometer, Vantaa, Finland), 25 µL of pollen extract (at final concentrations, 1250 1000, 750, 500, 250, 50 µg/mL) was mixed with 40 µL of tyrosinase (EC 1.14.1.8.1, 30 L, mushroom enzyme, Sigma Chemical Co.) and 100 µL of phosphate buffer, pH 6.8. This was then incubated at 37 °C for 15 min. Later, 40 µL of l-3.4-dihydroxyphenylalanine (l-dopa) (Sigma Chemical Co.) was added to the mixture and, after 10 min of incubation at 37 °C, absorbance was measured at 492 nm. The positive control was kojic acid (Sigma Chemical Co.). The percentage of inhibition of tyrosinase was calculated using the formula: % Inhibition = [(Abs_white_ − Abs_sample_)/Abs_white_] × 100. The extract concentration providing 50% inhibition (EC_50_) was obtained using the graph of I% against extract concentration.

#### 4.5.5. Hyaluronidase

Eighty U of hyaluronidase (Sigma Chemical Co.) was mixed with 100 µL of sodium phosphate buffer (20 mM/mL) and with 25 µL of bee pollen extract dissolved in DMSO (30 mg/mL) and incubated for 10 min at 37 °C, following the Sigma protocol [48]. After, we added 100 µL of hyaluronic acid (Sigma Chemical Co.) and the mixture was incubated at 37 °C for 45 min. The undigested hyaluronic acid was precipitated using 1 mL of albumin solution (albumin of bovine serum, at 0.1%, in sodium acetate 24 mM, Sigma Chemical Co.; and 79 mM of acetic acid, pH 3.75. This was placed to rest for 10 min at room temperature and absorbance was read at 600 nm. The reference for maximum inhibition was the absorbance value obtained in the absence of enzyme.

The inhibitory activity was measured as the percentage ratio of the absorbance in the presence of the test sample versus the reference value expressed by: Inhibition (%) = As/Aref × 100; As and Aref are the absorbance of the sample and absorbance of the reference, respectively.

#### 4.5.6. Lipoxygenase

To assess the effect on the enzyme lipoxygenase, the assay kit of Lipoxygenase inhibition (LOX) (Abnova, Walnut, CA, USA) was used. The hydroperoxides produced in lipoxygenation using a purified LOX were quantified at 490 nm. Nordihydroguaiaretic acid (NDGA, Abnova) was used as a negative control. The extract concentration providing 50% inhibition (EC_50_) was measured using the graph of I% against extract concentration, by interpolation.

#### 4.5.7. Antihemolytic Assay

The antihemolytic assay was performed as reported by Valente et al. [49]. A total of 10 mL of sheep blood was placed in a tube coated with citrate, which was centrifuged at 1500 r·min^−1^ at 4 °C for 10 min. Next, three washes were performed using phosphate buffered saline (PBS) (0.02 mol/L, pH of 7.4) and resuspended at 2% using PBS. In order to assess the protective effects of extracts/patterns against hemolysis induced by 2.2’-Azobis (2-amidinopropane) dihydrochloride (AAPH) (Sigma Chemical Co.), the erythrocyte suspension was pre-incubated with the extracts/patterns (50 μg of patterns and 50–200 μL of sample) at 37 °C for 30 min. Later, AAPH (dissolved in PBS, final concentration 50 mmol/L) was added and the mixture was gently stirred during incubation at 37 °C for 4 h. A negative control, containing erythrocytes in PBS, but also the controls of the extracts (erythrocytes in PBS with each extract), was used in all experiments. The hemolysis extent was measured at 540 nm (A) and compared with that of complete hemolysis (B) using distilled water (same volume of the reaction mixture) [50]. The percentage of hemolysis was obtained through: A/B × 100. Ascorbic acid (Merck) was used as reference antioxidant.

### 4.6. Statistical Analyzes

All experiments were carried out in triplicate. In order to assess significant differences between pollen samples, one-way analysis of variance (ANOVA) was used. As post-hoc test it was used the Scott-Knott test for all parameters apart from fatty-acids composition, where it was used the Tukey’s. Regression equations were estimated for phenol total and antioxidant activities, using the method of least squares. For all analyses, we adopted a significance level of 5*%* (*p* < 0.05). Moreover, we used principal components analysis to verify the relationship between variables and samples. The “R” statistical and programming environment version 3.0.2 (R Foundation, Vienna, Austria) was used [51].

## 5. Conclusions

This study provides additional information on the chemical properties and biological activities of different bee pollen samples. The studied samples constitute a nutritional source of phenolic compounds and health-promoting fatty acids, particularly PUFA, and possess low atherogenic and the thrombogenic indexes. Also, all samples presented important antioxidant activity, regardless of the assessment methodology, as well as moderate enzyme inhibition capacity.

In general, significant variation was found among the parameters of bee pollen from different plant genera. On the other hand, there was little variation in both chemical composition and biological activity of samples with the same botanical origin (which could be due to the geographical diversity).

Collectively, our results contribute to promoting the consumption of this traditional beehive product. Also, they provide some evidence regarding its potential for the prevention of health conditions in which free radicals and some enzymatic pathways are implicated. Further studies, using more samples, for different provenences and in more complex settings, must be conducted in order to confirm this hypothesis.

## Figures and Tables

**Table 1:** Fatty acid composition in pollen extracts (g/100 g of bee pollen).

Fatty Acid ^2^	Pollen Extracts (Mean ± SD) ^1^								
	S1	S2	S3	S4	S5	S6	S7	S8	S9
**C4:0**	0.035 ± 0.005 ^d^	0.021 ± 0.002 ^ab^	0.026 ± 0.002 ^bc^	0.040 ± 0.002 ^d^	0.037 ± 0.002 ^cd^	0.020 ± 0.002 ^ab^	0.074 ± 0.006 ^e^	0.019 ± 0.001 ^ab^	0.011 ± 0.001 ^a^
**C6:0**	0.036 ± 0.004 ^b^	0.040 ± 0.000 ^b^	0.039 ± 0.002 ^b^	0.043 ± 0.002 ^b^	0.038 ± 0.003 ^b^	0.037 ± 0.004 ^b^	0.070 ± 0.000 ^c^	0.025 ± 0.001 ^a^	0.043 ± 0.003 ^b^
**C8:0**	0.066 ± 0.001 ^ab^	0.051 ± 0.002 ^a^	0.052 ± 0.003 ^a^	0.093 ± 0.005 ^c^	0.110 ± 0.009 ^d^	0.066 ± 0.005 ^ab^	0.116 ± 0.005 ^d^	0.070 ± 0.001 ^b^	0.075 ± 0.003 ^b^
**C10:0**	0.015 ± 0.001 ^ab^	0.014 ± 0.001 ^ab^	0.013 ± 0.001 ^ab^	0.049 ± 0.001 ^cd^	0.055 ± 0.000 ^d^	0.042 ± 0.003 ^c^	0.067 ± 0.002 ^e^	0.010 ± 0.000 ^a^	0.019 ± 0.002 ^b^
**C12:0**	0.019 ± 0.001 ^b^	0.035 ± 0.004 ^d^	0.034 ± 0.003 ^d^	0.029 ± 0.001 ^cd^	0.029 ± 0.002 ^cd^	0.024 ± 0.002 ^bc^	0.050 ± 0.000 ^e^	0.010 ± 0.000 ^a^	0.006 ± 0.000 ^a^
**C14:0**	0.077 ± 0.002 ^e^	0.076 ± 0.003 ^e^	0.059 ± 0.002 ^d^	0.033 ± 0.002 ^bc^	0.031 ± 0.001 ^b^	0.042 ± 0.003 ^c^	0.083 ± 0.003 ^e^	0.017 ± 0.002 ^a^	0.011 ± 0.001 ^a^
**C16:0**	0.391 ± 0.036 ^a^	0.410 ± 0.051 ^a^	0.467 ± 0.029 ^a^	0.773 ± 0.016 ^c^	0.720 ± 0.009 ^c^	0.578 ± 0.022 ^b^	0.777 ± 0.045 ^c^	0.443 ± 0.017 ^a^	0.471 ± 0.005 ^a^
**C18:0**	0.050 ± 0.000 ^ab^	0.050 ± 0.000 ^ab^	0.050 ± 0.000 ^ab^	0.097 ± 0.005 ^c^	0.098 ± 0.006 ^c^	0.095 ± 0.003 ^c^	0.109 ± 0.011 ^c^	0.065 ± 0.004 ^b^	0.037 ± 0.002 ^a^
**C18:1n9**	0.388 ± 0.008 ^ab^	0.363 ± 0.023 ^ab^	0.425 ± 0.023 ^b^	0.632 ± 0.040 ^c^	0.610 ± 0.036 ^c^	0.639 ± 0.052 ^c^	0.950 ± 0.028 ^d^	0.447 ± 0.019 ^b^	0.336 ± 0.015 ^a^
**C18:3n3**	1.417 ± 0.042 ^b^	1.503 ± 0.082 ^bc^	1.417 ± 0.057 ^b^	1.750 ± 0.022 ^d^	1.707 ± 0.039 ^cd^	1.471 ± 0.111 ^b^	1.758 ± 0.082 ^d^	1.718 ± 0.045 ^d^	1.204 ± 0.046 ^a^
**C18:2n6c**	0.698 ± 0.056 ^ab^	0.782 ± 0.057 ^abc^	0.775 ± 0.063 ^abc^	0.877 ± 0.025 ^bcd^	0.930 ± 0.014 ^cd^	0.792 ± 0.049 ^abcd^	0.970 ± 0.078 ^d^	0.787 ± 0.041 ^abcd^	0.657 ± 0.018 ^a^
**Sums, Ratios and Indexes of Fatty Acids**									
**SFA**	0.694 ± 0.027 ^a^	0.696 ± 0.045 ^a^	0.739 ± 0.027 ^a^	1.158 ± 0.009 ^c^	1.116 ± 0.011 ^c^	0.904 ± 0.017 ^b^	1.345 ± 0.033 ^d^	0.655 ± 0.011 ^a^	0.673 ± 0.009 ^a^
**MUFA**	0.388 ± 0.008 ^ab^	0.363 ± 0.023 ^ab^	0.425 ± 0.0023 ^b^	0.632 ± 0.040 ^c^	0.610 ± 0.036 ^c^	0.639 ± 0.043 ^c^	0.0950 ± 0.028 ^d^	0.447 ± 0.019 ^b^	0.328 ± 0.024 ^a^
**PUFA**	2.116 ± 0.096 ^ab^	2.285 ± 0.127 ^bc^	2.192 ± 0.053 ^bc^	2.627 ± 0.033 ^d^	2.637 ± 0.037 ^d^	2.272 ± 0.105 ^bc^	2.728 ± 0.162 ^d^	2.504 ± 0.050 ^cd^	1.861 ± 0.060 ^a^
**NI**	0.106 ± 0.003 ^a^	0.123 ± 0.010 ^abc^	0.123 ± 0.005 ^abc^	0.193 ± 0.005 ^de^	0.223 ± 0.017 ^ef^	0.160 ± 0.02 ^cd^	0.245 ± 0.007 ^f^	0.112 ± 0.004 ^ab^	0.155 ± 0.023 ^bcd^
**TFA**	3.304 ± 0.114 ^ab^	3.468 ± 0.160 ^bc^	3.479 ± 0.047 ^bc^	4.610 ± 0.012 ^e^	4.586 ± 0.018 ^e^	3.975 ± 0.070 ^d^	5.268 ± 0.200 ^f^	3.719 ± 0.076 ^cd^	3.009 ± 0.013 ^a^
**PUFA:SFA**	3.049 ± 0.080 ^de^	3.234 ± 0.051 ^d^	2.970 ± 0.159 ^de^	2.268 ± 0.016 ^ab^	2.362 ± 0.013 ^ab^	2.029 ± 0.124 ^bc^	2.514 ± 0.130 ^a^	3.823 ± 0.046 ^f^	2.768 ± 0.124 ^cd^
**n6:n3**	0.492 ± 0.028 ^a^	0.520 ± 0.028 ^a^	0.549 ± 0.062 ^a^	0.287 ± 0.002 ^a^	0.545 ± 0.017 ^a^	0.551 ± 0.045 ^a^	0.539 ± 0.068 ^a^	0.458 ± 0.030 ^a^	0.546 ± 0.015 ^a^
**AI**	0.287 ± 0.002 ^cd^	0.283 ± 0.002 ^c^	0.281 ± 0.014 ^c^	0.287 ± 0.002 ^cd^	0.268 ± 0.003 ^bc^	0.315 ± 0.013 ^bc^	0.264 ± 0.014 ^d^	0.176 ± 0.006 ^a^	0.239 ± 0.008 ^b^
**TI**	0.076 ± 0.004 ^ab^	0.076 ± 0.004 ^ab^	0.086 ± 0.009 ^bc^	0.113 ± 0.001 ^d^	0.110 ± 0.001 ^d^	0.120 ± 0.007 ^cd^	0.102 ± 0.010 ^d^	0.066 ± 0.004 ^a^	0.087 ± 0.003 ^bc^

^1^ For each row, different letters (a–f) indicate significant differences (*p* < 0.05). Extracts: S1—*Coccus nucifera*; S2—*Cocos nucifera*; S3—*Miconia* spp.; S4—*Miconia* spp.; S5—*Miconia* spp.; S6—*Spondias* spp.; S7—*Myrcia* spp.; S8—*Eucalyptus* spp.; S9—*Eucalyptus* spp.; ^2^ Fatty acids: Butyric acid (C6:0); caproic acid (C6:0); caprylic acid (C8:0); capric acid (C10:0); lauric acid (C12:0); myristic acid (C14:0); palmitic acid (C16:0); stearic acid (C18:0); oleic acid (C18:1n9); α-linolenic acid (C18:3n3); linoleic acid (C18:2n6c); SFA: total saturated fatty acids; MUFA: total monounsaturated fatty acids; PUFA: total polyunsaturated fatty acids; NI: not identified; TFA: total fatty acids; n6: total ω-6 fatty acids; n3: total ω-3 fatty acids; AI: Atherogenic Index; TI: Thrombogenic Index.

**Table 2:** Mean values and standard deviations for antioxidant activities of the pollen extracts under study.

Extracts ^2^	Antioxidant Activities (Mean ± SD) ^1^			
	ABTS	DPPH	BCB	FRP
**S1**	2.12 ± 0.03 ^f^	2.52 ± 0.06 ^g^	0.93 ± 0.06 ^f^	1.82 ± 0.14 ^h^
**S2**	2.59 ± 0.16 ^e^	1.94 ± 0.17 ^h^	1.22 ± 0.14 ^f^	2.22 ± 0.20 ^g^
**S3**	1.23 ± 0.13 ^h^	4.46 ± 0.35 ^f^	2.47 ± 0.11 ^d^	3.36 ± 0.16 ^f^
**S4**	0.91 ± 0.05 ^i^	5.58 ± 0.17 ^d^	2.71 ± 0.19 ^d^	3.08 ± 0.05 ^f^
**S5**	1.58 ± 0.15 ^g^	5.15 ± 0.07 ^e^	1.98 ± 0.20 ^e^	3.73 ± 0.17 ^e^
**S6**	4.92 ± 0.18 ^b^	6.56 ± 0.27 ^c^	6.71 ± 0.34 ^a^	8.77 ± 0.23 ^a^
**S7**	5.73 ± 0.16 ^a^	7.99 ± 0.21 ^a^	5.18 ± 0.40 ^b^	7.20 ± 0.17 ^b^
**S8**	3.37 ± 0.34 ^d^	7.45 ± 0.11 ^b^	4.05 ± 0.29 ^c^	5.15 ± 0.09 ^c^
**S9**	4.06 ± 0.07 ^c^	6.66 ± 0.28 ^c^	4.89 ± 0.16 ^b^	4.53 ± 0.18 ^d^
**BHA**	0.09 ± 0.01	1.25 ± 0.02	1.23 ± 0.03	1.45 ± 0.03

^1^ Statistically significant differences (*p* < 0.05) are indicated by different lower case letters (a–i) within samples for each methodology; ^2^ S1—*Cocos nucifera*; S2—*Cocos nucifera*; S3—*Miconia* spp.; S4—*Miconia* spp.; S5—*Miconia* spp.; S6—*Spondias* spp.; S7—*Myrcia* spp.; S8—*Eucalyptus* spp.; S9—*Eucalyptus* spp. Antioxidant activities expressed as EC_50_ (mg/mL); BHA (buthylated hydroxyanisole).

**Table 3:** Enzyme inhibitory activities of bee pollen extracts (mean values and standard deviations).

Extract	Enzyme Inhibitory Activities (Mean ± SD) ^1^						
	α-AMY ^2^	AChE ^2^	TYR ^2^	LOX ^2^	LIP ^2^	HYAL ^3^	AHA ^3^
**S1**	1015.94 ± 12.16 ^a^	827.48 ± 21.94 ^b^	1140.44 ± 58.88 ^a^	312.74 ± 12.64 ^a^	4.16 ± 0.08 ^a^	17.33 ± 0.76 ^a^	74.13 ± 1.76 ^d^
**S2**	910.79 ± 12.37 ^b^	967.53 ± 17.57 ^a^	999.16 ± 9.98 ^b^	285.12 ± 11.77 ^b^	3.57 ± 0.16 ^b^	15.67 ± 0.76 ^b^	73.11 ± 2.61 ^d^
**S3**	488.02 ± 12.35 ^c^	150.12 ± 14.10 ^c^	627.09 ± 28.22 ^d^	92.99 ± 2.47 ^d^	2.66 ± 0.09 ^d^	13.00 ± 0.50 ^c^	78.29 ± 2.37 ^c^
**S4**	412.89 ± 12.25 ^d^	89.66 ± 8.28 ^e^	701.02 ± 11.30 ^c^	116.91 ± 8.24 ^c^	3.28 ± 0.21 ^c^	14.00 ± 0.66 ^c^	85.83 ± 0.24 ^a^
**S5**	372.08 ± 5.48 ^e^	121.50 ± 2.90 ^c^	519.60 ± 16.99 ^e^	84.38 ± 2.77 ^d^	2.33 ± 0.16 ^e^	14.17 ± 0.58 ^c^	81.91 ± 1.17 ^b^
**S6**	116.17 ± 13.64 ^f^	71.52 ± 3.58 ^f^	212.69 ± 22.95 ^f^	23.62 ± 2.16 ^f^	0.81 ± 0.06 ^h^	11.33 ± 0.58 ^d^	61.44 ± 1.12 ^e^
**S7**	50.81 ± 1.45 ^g^	3.93 ± 0.64 ^h^	No activity	13.51 ± 1.16 ^f^	1.34 ± 0.11 ^g^	7.00 ± 0.50 ^f^	43.85 ± 1.65 ^h^
**S8**	20.52 ± 0.82 ^h^	38.38 ± 2.47 ^g^	54.14 ± 7.43 ^g^	54.95 ± 1.61 ^e^	1.89 ± 0.15 ^f^	9.25 ± 0.66 ^e^	51.31 ± 1.67 ^g^
**S9**	16.44 ± 0.79 ^h^	26.85 ± 1.61 ^g^	45.88 ± 1.62 ^g^	45.20 ± 2.39 ^e^	1.56 ± 0.09 ^g^	10.25 ± 0.66 ^e^	55.70 ± 1.97 ^f^
**Control**	51.44 ± 0.81 Ascarbose	0.005 ± 0.001 Eserine	5.19 ± 0.29 Kojic acid	61.92 ± 2.97 NDGA	0.018 ± 0.0007 Orlistat	98.85 ± 0.53 Epigall	98.78 ± 0.53 Ascorbic acid

^1^ Within the same column, different letters (a–h) indicate significant differences (*p* < 0.05); Extracts: S1—*Cocos nucifera*; S2—*Cocos nucifera*; S3—*Miconia* spp.; S4—*Miconia* spp.; S5—*Miconia* spp.; S6—*Spondias* spp.; S7—*Myrcia* spp.; S8—*Eucalyptus* spp.; S9—*Eucalyptus* spp.; α-AMY, α-amylase; AChE, acetylcholinesterase; TYR, tyrosinase; LOX, lipoxygenase; LIP, lipase; HYAL, hyaluronidase; AHA, anti-hemolytic activity; and ^2^ Expressed as EC_50_ (µg/mL); and ^3^ Expressed as % (concentration 150 μg/mL).

**Table 4:** Pollen types identified in samples of *A. mellifera*, and predominant botanical origin and harvesting site.

Pollen Extracts	Pollen Types *	Predominant Botanical Origin	Harvest Place/State
**S1**	**DP:** *Cocos nucifera* (100%)	*Cocos nucifera*	Neópolis/Sergipe
**S2**	**DP:** *Cocos nucifera* (100%)	*Cocos nucifera*	Maraú/Bahia
**S3**	**DP:** *Miconia* spp. (97.1%) **OIP:** *Cocos nucifera* (2.9%)	*Miconia* spp.	Valença/Bahia
**S4**	**DP:** *Miconia* spp. (98.5%) **OIP:** *Cocos nucifera* (1.5%)	*Miconia* spp.	Valença/Bahia
**S5**	**DP:** *Miconia* spp. (97.2%) **OIP:** *Cocos nucifera* (2.8%)	*Miconia* spp.	Valença/Bahia
**S6**	**DP:** *Spondias* spp. (95.8%) **IIP:** *Cocos nucifera* (4.2%)	*Spondias* spp.	Canavieiras/Bahia
**S7**	**DP:** *Myrcia* spp. (60.0%) **AP:** *Cocos nucifera* (33.3%) **IIP:** *Saccharum* spp. (6.7%)	Multifloral	Ilhéus/Bahia
**S8**	PD: *Eucalyptus* spp. (96.8%) **OIP:** *Mikania* spp. (2.2%), *Cocos nucifera* (1.0%)	*Eucalyptus* spp.	Teixeira de Freitas/Bahia
**S9**	**DP:** *Eucalyptus* spp. (97.0%) **OIP:** *Mimosa* spp. (1.0%), *Myrcia* spp. (2.0%)	*Eucalyptus* spp.	Canavieiras/Bahia

* DP = Dominant pollen (>45% of the pollen grains); AP = Accessory pollen (from 16% to 45% of the pollen grains); IIP = Important Isolated pollen (from 3% to 15%); OIP = Occasional Isolated pollen (<3%).
